# Supplementary material for: Dipeptidylpeptidase 4 inhibition attenuates gestational pathologies via immune homeostasis restoration in the pulmonary-uterine axis
Source: Nat Commun. 2026 Feb 17;17:2851. doi: 10.1038/s41467-026-69620-9 (PMC13021972; doi:10.1038/s41467-026-69620-9)
Supplement: Supplementary file 1 — Supplementary Information [file 41467_2026_69620_MOESM1_ESM.pdf]

Supplementary Information

Dipeptidylpeptidase 4 inhibition attenuates gestational pathologies via immune homeostasis restoration in the pulmonary-uterine axis

Guirong Shi[1], Shengdi Xi[1], Mengyuan Lv[1], Yihang Chen[1], Yonggang Zhou[1],
Haiming Wei[1]*, Binqing Fu[1]*

1. State Key Laboratory of Immune Response and Immunotherapy, Department of Obstetrics and Gynecology, The First Affiliated Hospital of USTC, Center for Advanced Interdisciplinary Science & Biomedicine of IHM, Division of Life Sciences and Medicine, University of Science and Technology of China; Hefei, Anhui, 230001, China.

*Correspondence: ustcwhm@ustc.edu.cn (H.W.) or fbq@ustc.edu.cn (B.F.)

The PDF file contains:

Supplementary Fig. 1-10:

Supplementary Fig. 1. Respiratory influenza virus infection during early pregnancy causes lung inflammation and intrauterine growth restriction, related to Fig. 1.

Supplementary Fig. 2. Influenza viral replication in the lung and decidua and pulmonary immune cells infiltration following sitagliptin treatment, related to Fig. 2.

Supplementary Fig. 3. Inhibition of DPP4 meliorates the compromised litter size caused by respiratory influenza virus infection, related to Fig. 2.

Supplementary Fig. 4. Inhibition of DPP4 also alleviates intrauterine growth restriction caused by respiratory influenza virus infection after placentation, related to Fig. 2.

Supplementary Fig. 5. Inhibition of DPP4 restores immune homeostasis in uterus and lung, related to Fig. 2.

Supplementary Fig. 6. Differential alterations in myeloid cell populations in maternal lungs, peripheral blood and deciduas following influenza infection, related to Fig. 4.

Supplementary Fig. 7. A single-cell atlas of the maternal immune response to influenza infection in the lung and decidua, related to Fig. 5.

Supplementary Fig. 8. Influenza viral replication in the lung and decidua following knocking out *Il1r2*, related to Fig. 6.

Supplementary Fig. 9. Intranasal MHV inoculation during early pregnancy causes lung inflammation and intrauterine growth restriction, related to Fig. 7.

Supplementary Fig. 10. Intranasal MHV inoculation results in the accumulation of IL1R2 and inhibition of DPP4 alleviates lung inflammation, related to Fig. 7.

Supplementary Table. 1-4:

Supplementary Table 1. PCR primers used for genotyping of *Il1r2*$^{-/-}$ mice, related to the methods.

Supplementary Table 2. Primers used for real-time quantitative PCR, related to the methods.

Supplementary Table 3. Antibodies used for flow cytometry analysis, related to the methods.

Supplementary Table 4. Antibodies used for immunohistochemistry and immunofluorescence, related to the methods.

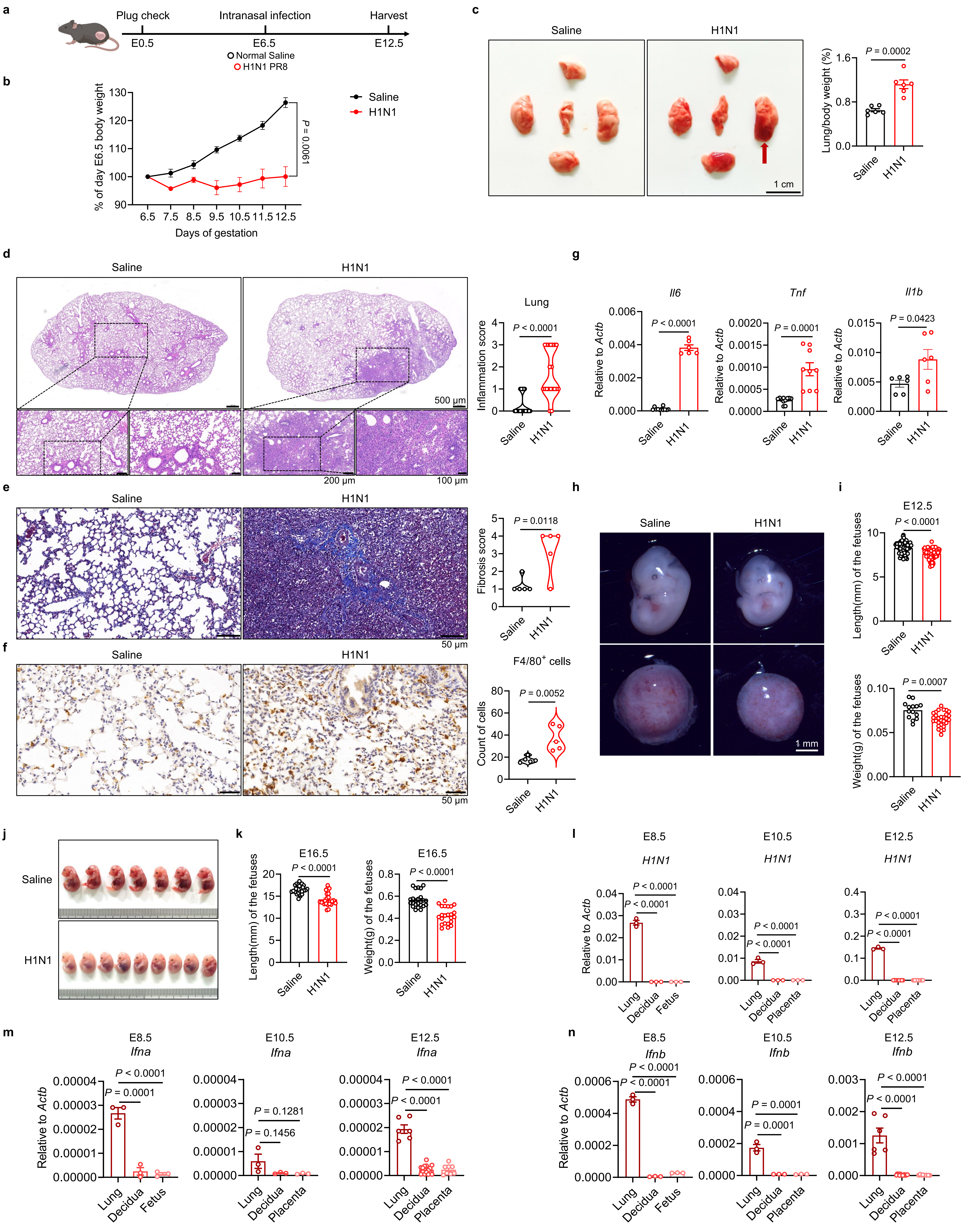

a
Plug check
Intranasal infection
Harvest
E0.5
E6.5
E12.5
Normal Saline
H1N1 PR8
b
% of day E6.5 body weight
Days of gestation
Saline
H1N1
P = 0.0061
c
Saline
H1N1
1 cm
Lung/body weight (%)
P = 0.0002
d
Saline
H1N1
500 μm
200 μm
100 μm
Lung
Inflammation score
P < 0.0001
e
Saline
H1N1
50 μm
Fibrosis score
P = 0.0118
f
Saline
H1N1
50 μm
F4/80+ cells
Count of cells
P = 0.0052
g
Il6
Tnf
Il1b
Relative to Actb
P < 0.0001
P = 0.0001
P = 0.0423
h
Saline
H1N1
1 mm
i
E12.5
Length(mm) of the fetuses
P < 0.0001
Weight(g) of the fetuses
P = 0.0007
j
Saline
H1N1
k
E16.5
Length(mm) of the fetuses
P < 0.0001
Weight(g) of the fetuses
P < 0.0001
l
E8.5
E10.5
E12.5
H1N1
Relative to Actb
Lung
Decidua
Fetus
Placenta
P < 0.0001
m
E8.5
E10.5
E12.5
Ifna
Relative to Actb
P < 0.0001
P = 0.0001
P = 0.1281
P = 0.1456
n
E8.5
E10.5
E12.5
Ifnb
Relative to Actb
P < 0.0001
P = 0.0001

Supplementary Fig. 1 | Respiratory influenza virus infection during early pregnancy causes lung inflammation and intrauterine growth restriction, related to Fig. 1. a Schematic diagram illustrating the timeline of H1N1 infection in pregnant mice (Created in BioRender. Ding, X. (2026) https://BioRender.com/lfokc6w). **b** Body weight changes in pregnant mice infected with either saline (*n* = 6) or H1N1 (*n* = 6). **c-g** Lungs were collected at embryonic day 12.5 (E12.5) for analysis. **c** Representative images of lungs infected with either saline (*n* = 6) or H1N1 (*n* = 6) (left). The ratio of lung tissue mass to mouse body weight (right). scale bar, 1 cm. The red arrow marks the parenchymal lung lesion. **d** Representative images of hematoxylin and eosin (H&E) staining of lung sections infected with either saline or H1N1. scale bar, 100 μm (left). Inflammation score for lung sections (right). **e** Representative images of Masson trichrome staining of lung sections infected with either saline or H1N1. scale bar, 50 μm (left). Fibrosis score for lung sections (right). **f** Representative images of immunohistochemistry staining of F4/80-positive cells in lung sections infected with either saline or H1N1. scale bar, 50 μm (left). Statistical analysis of F4/80-positive cells (right). **g** Quantitative PCR (qPCR) analysis of *Il6*, *Tnf*, and *Il1b* expression in lungs infected with either saline (*n* = 11 for *Il6*, *n* = 11 for *Tnf* and *n* = 6 for *Il1b*) or H1N1 (*n* = 6 for *Il6*, *n* = 9 for *Tnf* and *n* = 6 for *Il1b*). **h, i** Representative images of fetuses and placentas (**h**) and statistical analysis of fetal length and weight (**i**) in pregnant mice infected with either saline (*n* = 75 for fetal length and *n* = 14 for fetal weight) or H1N1 (*n* = 63 for fetal length and *n* = 25 for fetal weight) at E12.5. scale bar, 1 mm. **j, k** Representative images of fetuses (**j**) and statistical analysis of fetal length and weight (**k**) in pregnant mice infected with either saline (*n* = 28 for fetal length and *n* = 24 for fetal weight) or H1N1 (*n* = 26 for fetal length and *n* = 23 for fetal weight) at E16.5. **l-n** qPCR analysis of *H1N1* (*influenza matrix gene*), *Ifna* and *Ifnb* expression in lungs, deciduas, and placentas infected with H1N1 at E8.5 (*n* = 3 for *H1N1*, *n* = 3 for *Ifna*, *n* = 3 for *Ifnb* in lungs and *n* = 3 for *H1N1*, *n* = 3 for *Ifna*, *n* = 3 for *Ifnb* in deciduas and *n* = 3 for *H1N1*, *n* = 3 for *Ifna*, *n* = 3 for *Ifnb* in placentas), E10.5 *n* = 3 for *H1N1*, *n* = 3 for *Ifna*, *n* = 3 for *Ifnb* in lungs and *n* = 3 for *H1N1*, *n* = 3 for *Ifna*, *n* = 3 for *Ifnb* in deciduas and *n* = 3 for *H1N1*, *n* = 3 for *Ifna*, *n* = 3 for *Ifnb* in placentas), and E12.5 (*n* = 3 for *H1N1*, *n* = 3 for *Ifna*, *n* = 6 for *Ifnb* in lungs and *n* = 18 for *H1N1*, *n* = 15 for *Ifna*, *n* = 15 for *Ifnb* in deciduas and *n* = 18 for *H1N1*, *n* = 8 for *Ifna*, *n* = 9 for *Ifnb* in placentas). Results are representative of two or three independent experiments. All bars in the graphs represent the mean ± s.e.m. Statistical comparisons were performed using a two-tailed unpaired Student's t-test (**b-k**) and one-way analysis of variance (ANOVA) with Dunnett's multiple comparisons test (**l-n**). Source data are provided as a Source Data file.

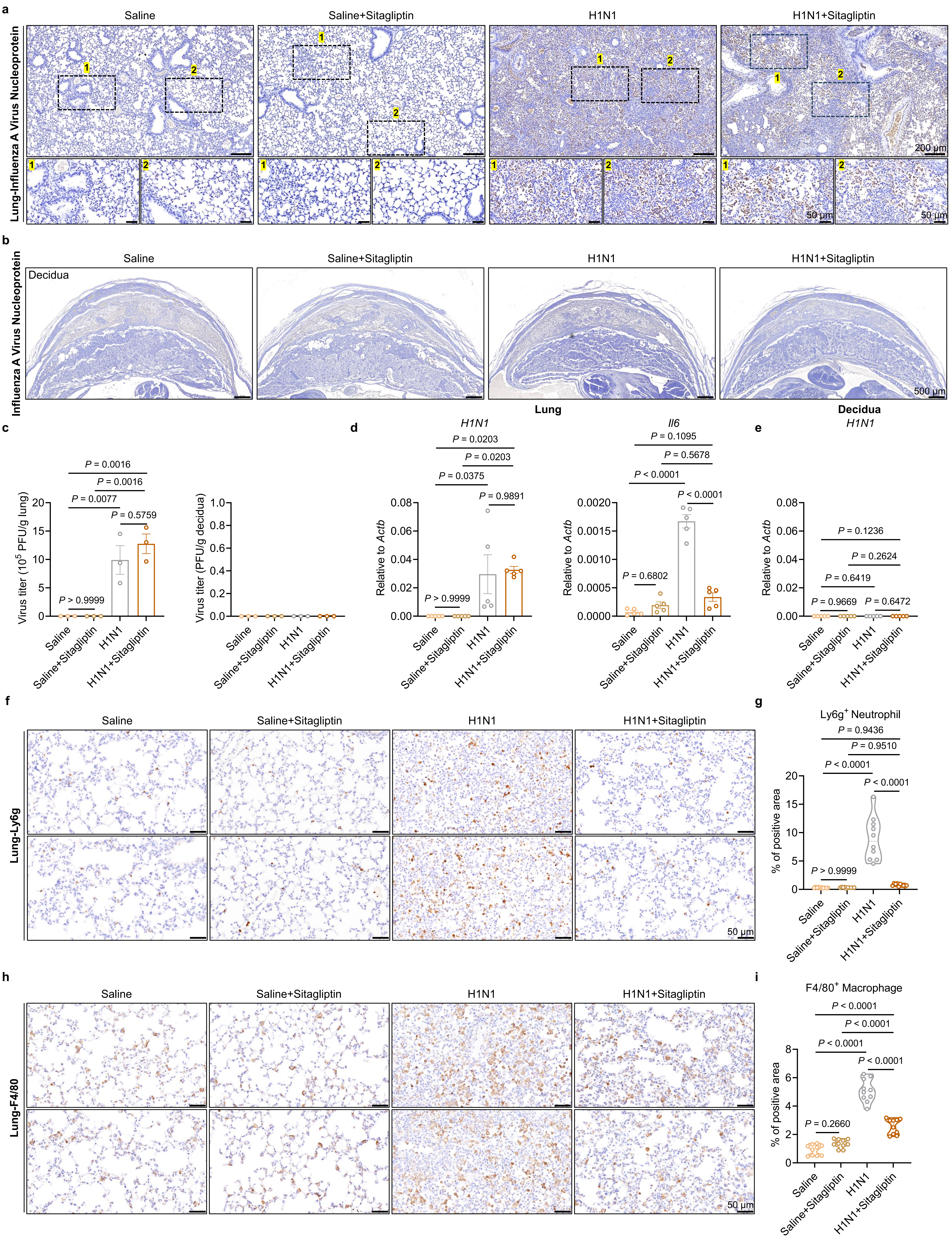

a
Saline
Saline+Sitagliptin
H1N1
H1N1+Sitagliptin
Lung-Influenza A Virus Nucleoprotein
200 μm
50 μm
b
Influenza A Virus Nucleoprotein
Decidua
500 μm
c
Virus titer (10^5 PFU/g lung)
P = 0.0016
P = 0.0016
P = 0.0077
P = 0.5759
P > 0.9999
Virus titer (PFU/g decidua)
d
Lung
H1N1
P = 0.0203
P = 0.0203
P = 0.0375
P = 0.9891
P > 0.9999
Relative to Actb
Il6
P = 0.1095
P = 0.5678
P < 0.0001
P < 0.0001
P = 0.6802
e
Decidua
H1N1
P = 0.1236
P = 0.2624
P = 0.6419
P = 0.9669
P = 0.6472
f
Lung-Ly6g
50 μm
g
Ly6g+ Neutrophil
P = 0.9436
P = 0.9510
P < 0.0001
P < 0.0001
P > 0.9999
% of positive area
h
Lung-F4/80
50 μm
i
F4/80+ Macrophage
P < 0.0001
P < 0.0001
P < 0.0001
P < 0.0001
P = 0.2660
% of positive area

Supplementary Fig. 2 | Influenza viral replication in the lung and decidua and pulmonary immune cells infiltration following sitagliptin treatment, related to Fig. 2. a-i Maternal lungs and deciduas were collected at embryonic day 12.5 (E12.5) for analysis. **a** Representative images of immunohistochemistry staining of Influenza A Virus Nucleoprotein in lung sections infected with saline alone or treated with sitagliptin, and infected with H1N1 alone or treated with sitagliptin. scale bar, 50 µm. **b** Representative images of immunohistochemistry staining of Influenza A Virus Nucleoprotein in decidua sections infected with saline alone or treated with sitagliptin, and infected with H1N1 alone or treated with sitagliptin. scale bar, 500 µm. **c** Virus titers were determined in lungs and deciduas infected with saline alone (n = 3) or treated with sitagliptin (n = 3), and infected with H1N1 alone (n = 3) or treated with sitagliptin (n = 3). **d** qPCR analysis of *H1N1* (*influenza matrix gene*) and *Il6* expression in lungs infected with saline alone (n = 5) or treated with sitagliptin (n = 5), and infected with H1N1 alone (n = 5) or treated with sitagliptin (n = 5). **e** qPCR analysis of *H1N1* (*influenza matrix gene*) expression in deciduas infected with saline alone (n = 5) or treated with sitagliptin (n = 5), and infected with H1N1 alone (n = 5) or treated with sitagliptin (n = 5). **f, g** Representative images of immunohistochemistry staining of Ly6g-positive cells in lung sections infected with saline alone (n = 10) or treated with sitagliptin (n = 10), and infected with H1N1 alone (n = 10) or treated with sitagliptin (n = 10) (**f**) and quantification of Ly6g positive staining area (as the percentage of the total area) by ImageJ (**g**). scale bar, 50 µm. **h, i** Representative images of immunohistochemistry staining of F4/80-positive cells in lung sections infected with saline alone (n = 10) or treated with sitagliptin (n = 10), and infected with H1N1 alone (n = 10) or treated with sitagliptin (n = 10) (**h**) and quantification of F4/80 positive staining area (as the percentage of the total area) by ImageJ (**i**). scale bar, 50 µm. All bars in the graphs represent the mean ± s.e.m. Statistical comparisons were performed using one-way ANOVA with Tukey's multiple comparisons test (**c-e, g** and **i**). Source data are provided as a Source Data file.

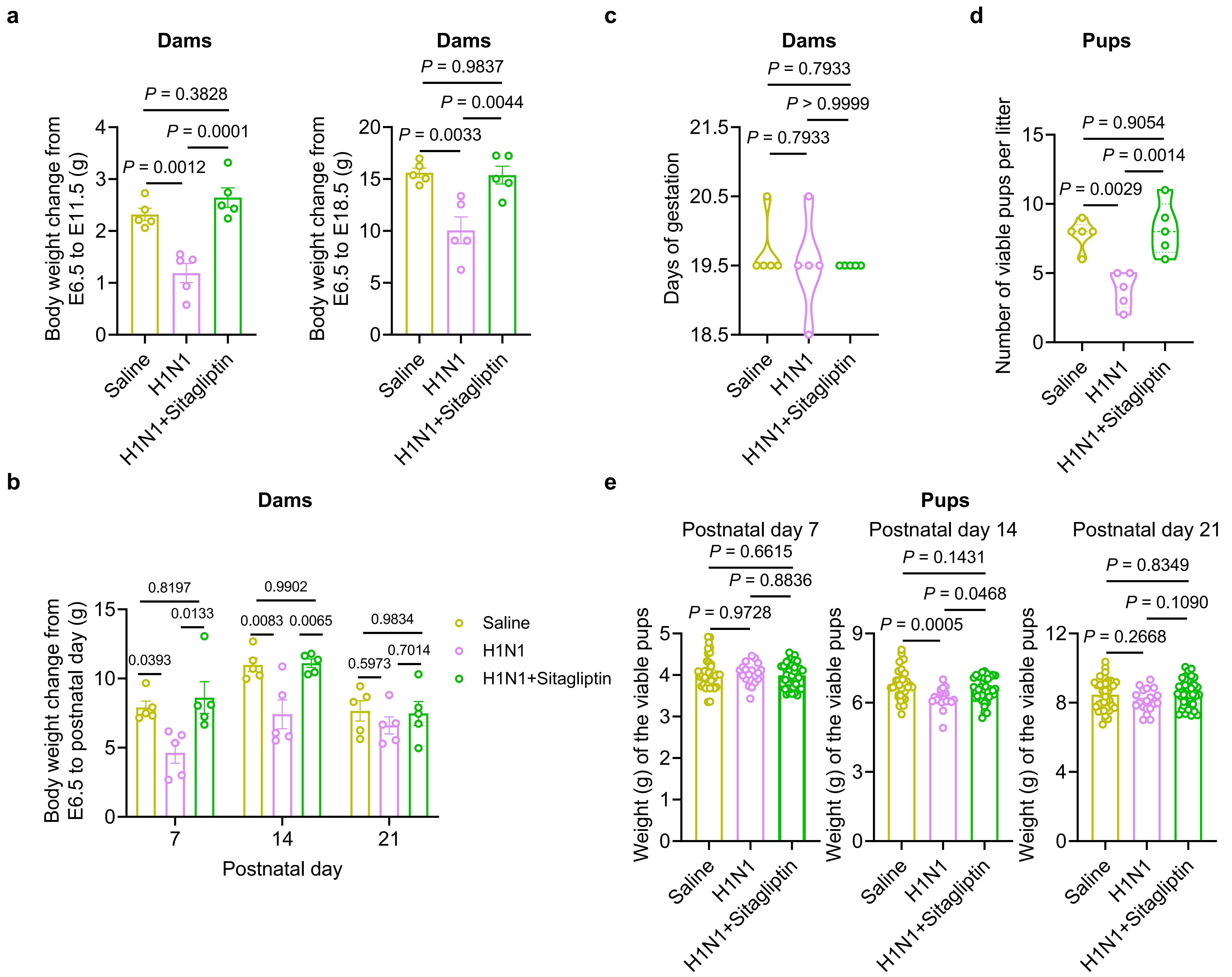

Supplementary Fig. 3 | Inhibition of DPP4 meliorates the compromised litter size caused by respiratory influenza virus infection, related to Fig. 2. a, b Body weight change from embryonic day 6.5 (E6.5) to E11.5 and to E18.5 of dams infected with either saline (*n* = 5) or H1N1 alone (*n* = 5), or treated with sitagliptin (*n* = 5). **b** Body weight change from embryonic day 6.5 (E6.5) to postnatal day 7, day14 and day 21 of dams infected with either saline (*n* = 5) or H1N1 alone (*n* = 5), or treated with sitagliptin (*n* = 5). **c** Days of gestation of dams infected with either saline (*n* = 5) or H1N1 alone (*n* = 5), or treated with sitagliptin (*n* = 5). **d** Number of viable pups per litter delivered by dams infected with either saline (*n* = 5) or H1N1 alone (*n* = 5), or treated with sitagliptin (*n* = 5). **e** Weight of the viable pups at postnatal day 7, day14 and day 21 delivered by dams infected with either saline (*n* = 39) or H1N1 alone (*n* = 19), or treated with sitagliptin (*n* = 5). All bars in the graphs represent the mean ± s.e.m. Statistical comparisons were performed using one-way ANOVA with Tukey's multiple comparisons test (**a-e**). Source data are provided as a Source Data file.

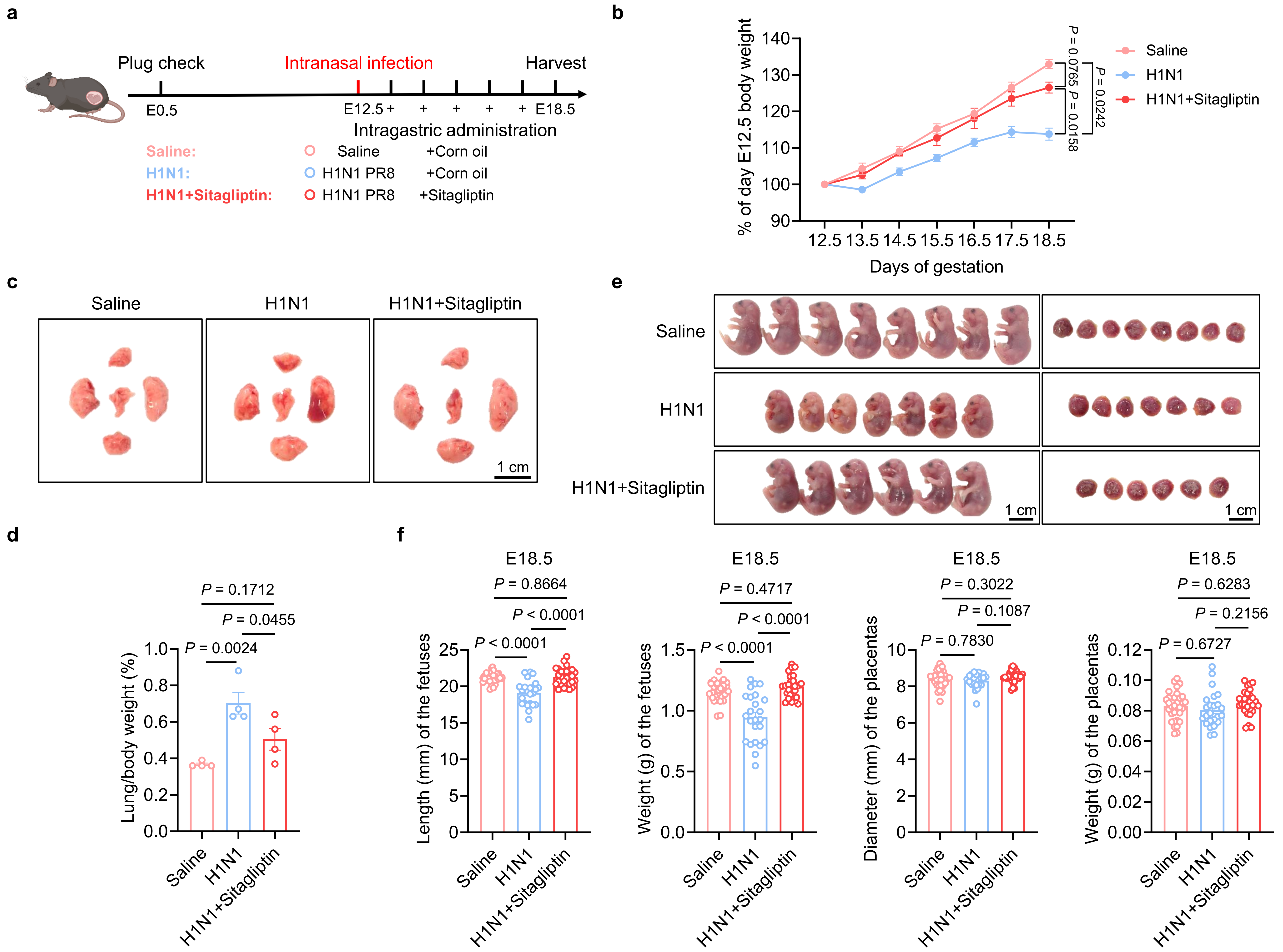

Supplementary Fig. 4 | Inhibition of DPP4 also alleviates intrauterine growth restriction caused by respiratory influenza virus infection after placentation, related to Fig. 2. a Schematic diagram illustrating the timeline of H1N1 infection and sitagliptin treatment in pregnant mice (Created in BioRender. Ding, X. (2026) https://BioRender.com/lfokc6w). **b** Body weight changes in pregnant mice infected with saline (n = 4) or infected with H1N1 alone (n = 4), or treated with sitagliptin (n = 4). **c-f** Lungs, fetuses and placentas were collected at embryonic day 18.5 (E18.5) for analysis. **c, d** Representative images of lungs infected with either saline (n = 4) or H1N1 alone (n = 4), or treated with sitagliptin (n = 4) (**c**). The ratio of lung tissue mass to mouse body weight (**d**). scale bar, 1 cm. **e, f** Representative images of fetuses and placentas (**e**) and statistical analysis of the length and weight of fetuses and the diameter and weight of placentas (**f**) infected with either saline (n = 31 for length of fetuses, n = 31 for weight of fetuses, n = 31 for diameter of placentas and n = 31 for weight of placentas) or H1N1 alone (n = 24 for length of fetuses, n = 24 for weight of fetuses, n = 24 for diameter of placentas and n = 24 for weight of placentas), or treated with sitagliptin (n = 27 for length of fetuses, n = 27 for weight of fetuses, n = 27 for diameter of placentas and n = 27 for weight of placentas). scale bar, 1 cm. All bars in the graphs represent the mean ± s.e.m. Statistical comparisons were performed using one-way ANOVA with Tukey's multiple comparisons test (**b, d** and **f**). Source data are provided as a Source Data file.

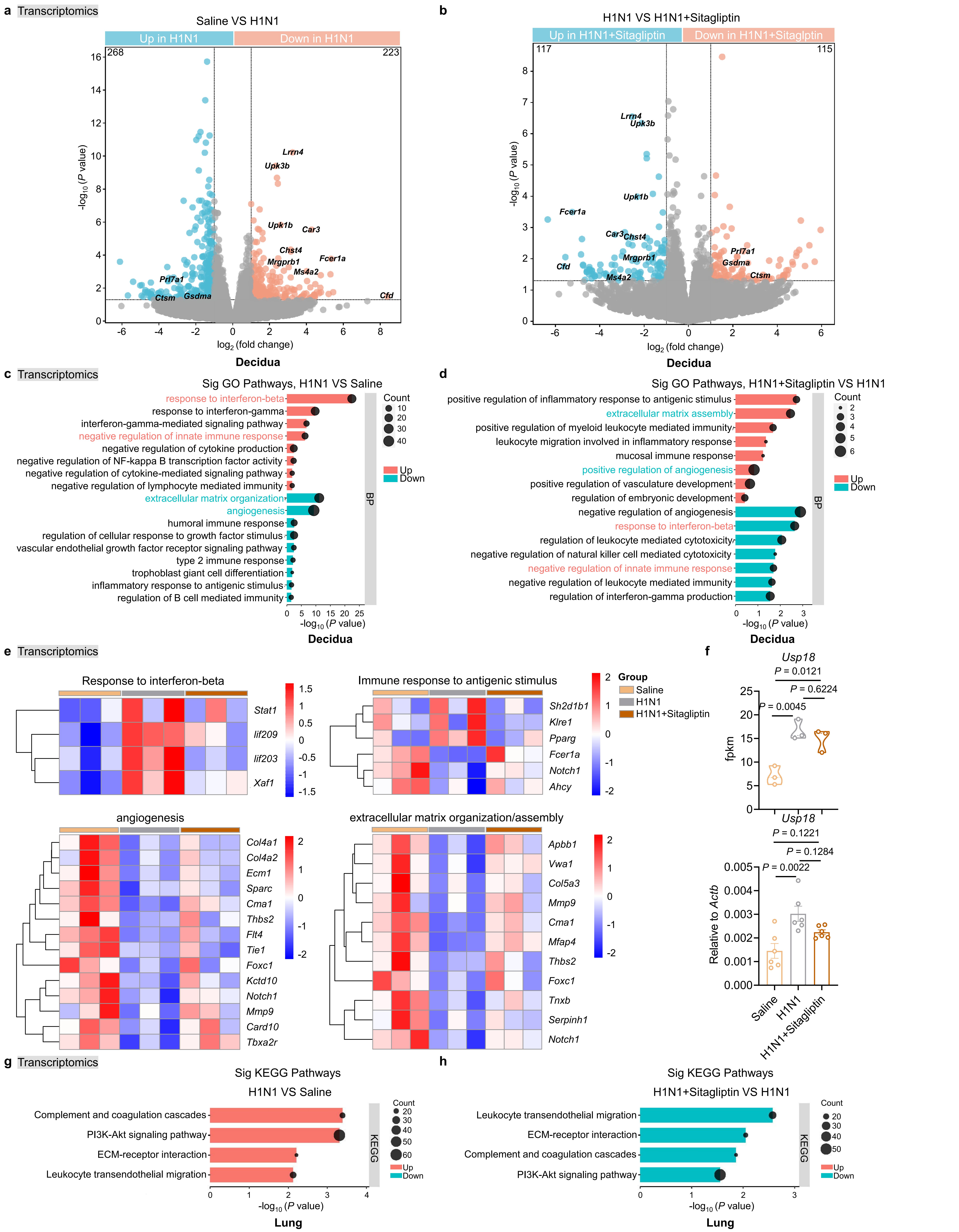

Supplementary Fig. 5 | Inhibition of DPP4 restores immune homeostasis in uterus and lung, related to Fig. 2. a-f Maternal deciduas were collected at embryonic day 12.5 (E12.5) for analysis. **a, b** Transcriptomic analysis of differentially expressed genes in deciduas infected with either saline ($n = 3$) or H1N1 alone ($n = 3$), or treated with sitagliptin ($n = 3$). **c, d** Gene Ontology (GO) of Biological Process (BP) analysis of differentially expressed genes in deciduas infected with either saline ($n = 3$) or H1N1 alone ($n = 3$), or treated with sitagliptin ($n = 3$). **e** Gene expression heatmap for angiogenesis and immune responses to stimulus in deciduas infected with either saline ($n = 3$) or H1N1 alone ($n = 3$), or treated with sitagliptin ($n = 3$). **f** Fpkm values and qPCR analysis of *Usp18* expression in deciduas infected with either saline ($n = 6$) or H1N1 ($n = 6$), or treated with sitagliptin ($n = 6$). **g-h** Maternal lungs were collected at embryonic day 12.5 (E12.5) for analysis. **g, h** Kyoto Encyclopedia of Genes and Genomes (KEGG) pathway enrichment analysis of lungs infected with either saline ($n = 3$) or H1N1 alone ($n = 3$), or treated with sitagliptin ($n = 3$). Results are representative of two or three independent experiments. All bars in the graphs represent the mean ± s.e.m. Statistical comparisons were performed using one-way ANOVA with Tukey's multiple comparisons test (**f**). Source data are provided as a Source Data file.

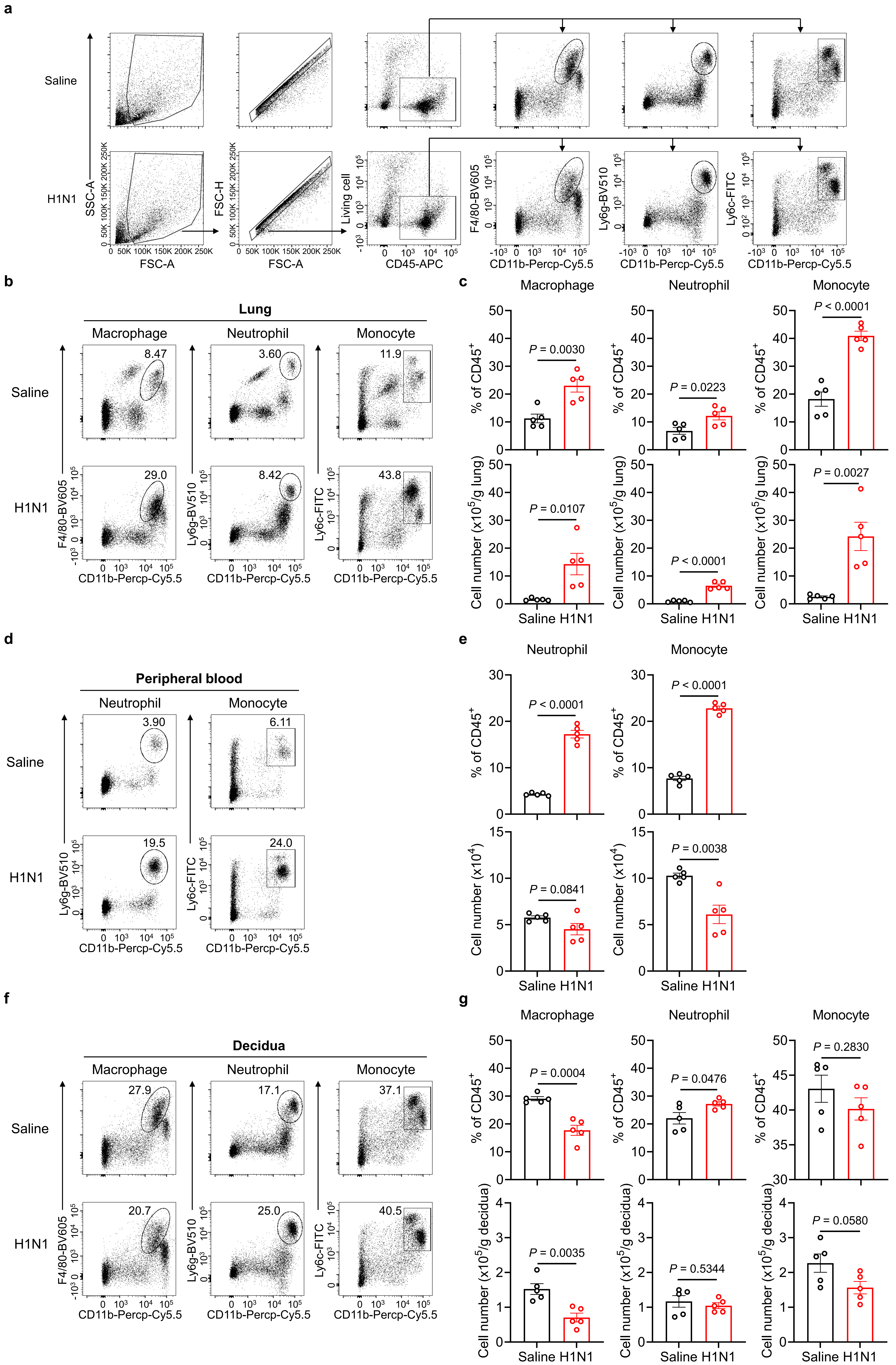

Supplementary Fig. 6 | Differential alterations in myeloid cell populations in maternal lungs, peripheral blood and deciduas following influenza infection, related to Fig. 4. a-g Maternal lungs, peripheral blood and deciduas were collected at embryonic day 12.5 (E12.5) for analysis. **a** Gating strategy for the identification of macrophages, neutrophils and monocytes in the lung, peripheral blood, and decidua infected with either saline or H1N1 presented on Fig 4a-e. **b, c** Representative flow cytometry dot plots (**b**) and analysis of the percentage and cell number of macrophages, neutrophils and monocytes (**c**) in lungs infected with either saline (n = 5) or H1N1 (n = 5). **d, e** Representative flow cytometry dot plots (**d**) and analysis of the percentage and cell number of neutrophils and monocytes (**e**) in peripheral blood infected with either saline (n = 5) or H1N1 (n = 5). **f, g** Representative flow cytometry dot plots (**f**) and analysis of the percentage and cell number of macrophages, neutrophils and monocytes (**g**) in deciduas infected with either saline (n = 5) or H1N1 (n = 5). All bars in the graphs represent the mean ± s.e.m. Statistical comparisons were performed using a two-tailed unpaired Student's t-test (**c**, **e** and **g**). Source data are provided as a Source Data file.

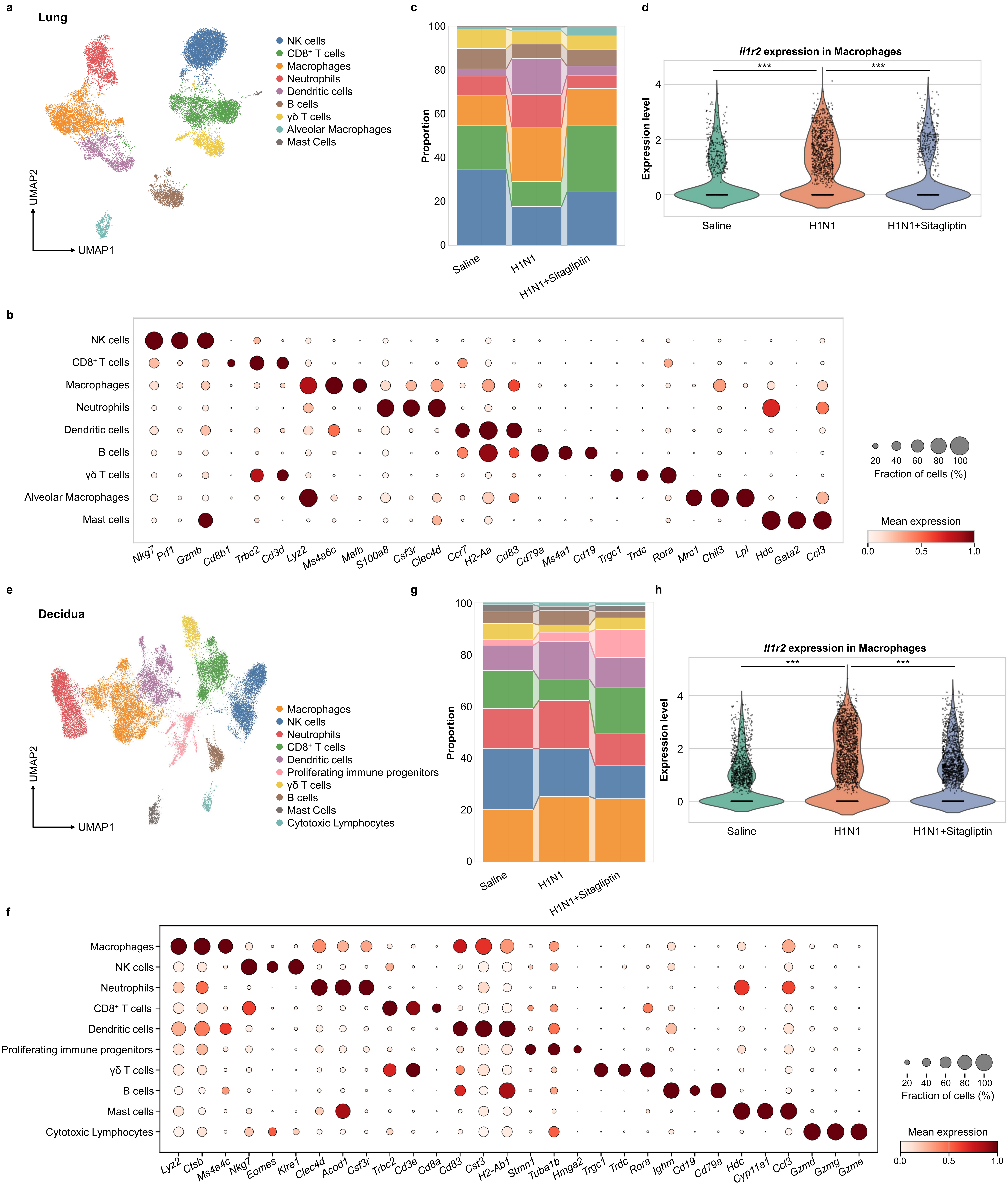
a
Lung
NK cells
CD8⁺ T cells
Macrophages
Neutrophils
Dendritic cells
B cells
γδ T cells
Alveolar Macrophages
Mast Cells
UMAP2
UMAP1
c
Proportion
Saline
H1N1
H1N1+Sitagliptin
d
Il1r2 expression in Macrophages
Expression level
***
Saline
H1N1
H1N1+Sitagliptin
b
Nkg7 Prf1 Gzmb Cd8b1 Trbc2 Cd3d Lyz2 Ms4a6c Mafb S100a8 Csf3r Clec4d Ccr7 H2-Aa Cd83 Cd79a Ms4a1 Cd19 Trgc1 Trdc Rora Mrc1 Chil3 Lpl Hdc Gata2 Ccl3
Fraction of cells (%)
Mean expression
e
Decidua
Macrophages
NK cells
Neutrophils
CD8⁺ T cells
Dendritic cells
Proliferating immune progenitors
γδ T cells
B cells
Mast Cells
Cytotoxic Lymphocytes
g
h
f
Lyz2 Ctsb Ms4a4c Nkg7 Eomes Klre1 Clec4d Acod1 Csf3r Trbc2 Cd3e Cd8a Cd83 Cst3 H2-Ab1 Stmn1 Tuba1b Hmga2 Trgc1 Trdc Rora Ighm Cd19 Cd79a Hdc Cyp11a1 Ccl3 Gzmd Gzmg Gzme

Supplementary Fig. 7 | A single-cell atlas of the maternal immune response to influenza infection in the lung and decidua, related to Fig. 5. a-d Maternal lungs were collected at embryonic day 12.5 (E12.5) for analysis. **a** Uniform manifold approximation and projection (UMAP) plot showing the cell types of lungs infected with saline or infected with H1N1 alone, or treated with sitagliptin. **b** Dot plots of normalized expression of selected cluster-specific genes in lungs. Three genes are shown per cluster. **c** Proportion of each cluster in lungs infected with either saline or H1N1 alone, or treated with sitagliptin. **d** Violin plots of *Il1r2* expression in macrophages in lungs infected with either saline or H1N1 alone, or treated with sitagliptin. *** represents $P < 0.001$. **e-h** Maternal deciduas were collected at embryonic day 12.5 (E12.5) for analysis. **e** UMAP plot showing the cell types of deciduas infected with saline or infected with H1N1 alone, or treated with sitagliptin. **f** Dot plots of normalized expression of selected cluster-specific genes in deciduas. **g** Proportion of each cluster in deciduas infected with either saline or H1N1 alone, or treated with sitagliptin. **h** Violin plots of *Il1r2* expression in macrophages in deciduas infected with either saline or H1N1 alone, or treated with sitagliptin. *** represents $P < 0.001$.

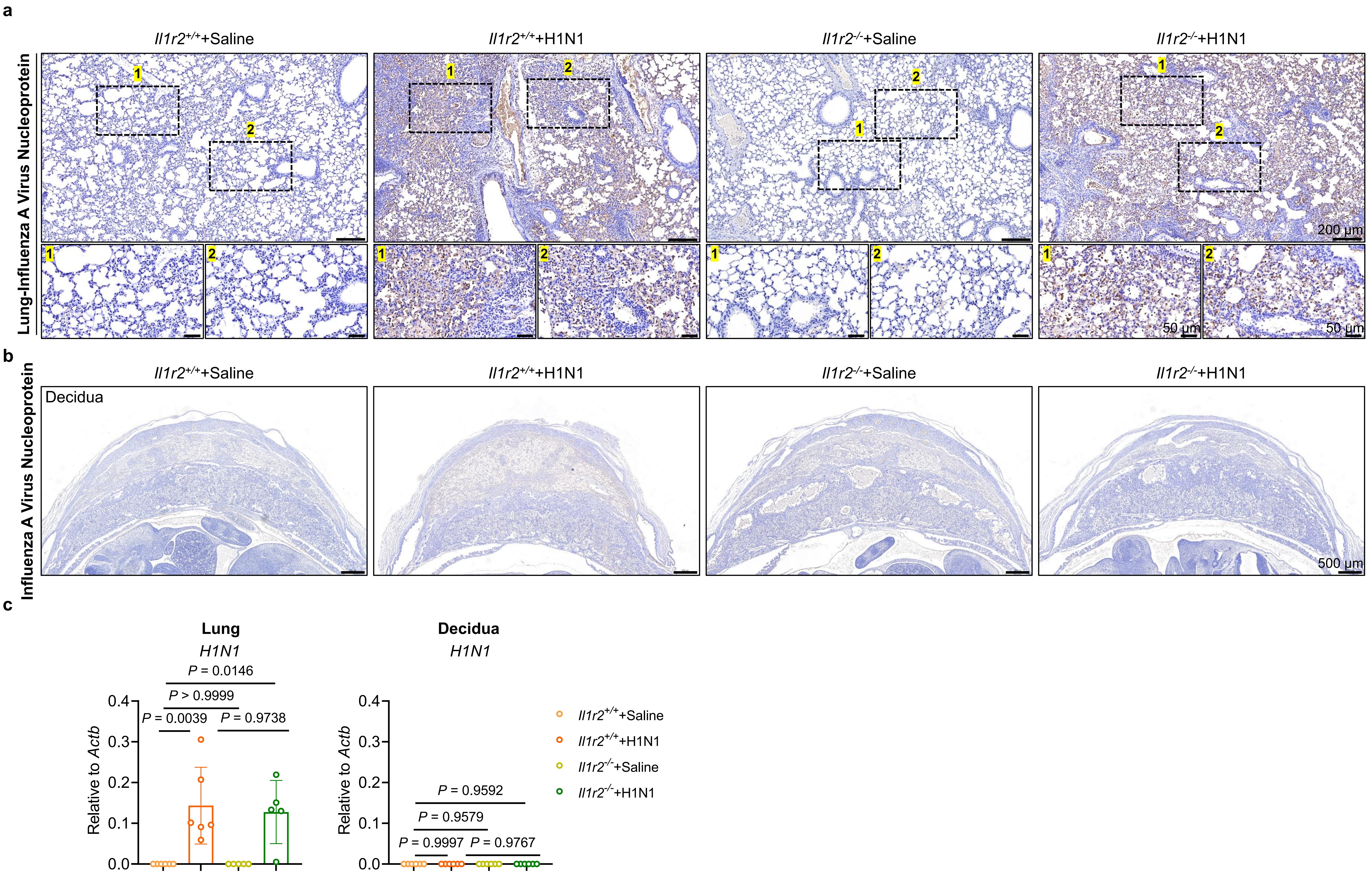

Supplementary Fig. 8 | Influenza viral replication in the lung and decidua following knocking out *Il1r2*, related to Fig. 6. a-c Maternal lungs and deciduas were collected at embryonic day 12.5 (E12.5) for analysis. **a** Representative images of immunohistochemistry staining of Influenza A Virus Nucleoprotein in lung sections from pregnant *Il1r2*$^{+/+}$ and *Il1r2*$^{-/-}$ mice infected with either saline or H1N1. scale bar, 50 μm. **b** Representative images of immunohistochemistry staining of Influenza A Virus Nucleoprotein in decidua sections from pregnant *Il1r2*$^{+/+}$ and *Il1r2*$^{-/-}$ mice infected with either saline or H1N1. scale bar, 500 μm. **c** qPCR analysis of *H1N1* (*influenza matrix gene*) expression in lungs and deciduas from pregnant *Il1r2*$^{+/+}$ (n = 6 for Saline and n = 6 for *H1N1*) and *Il1r2*$^{-/-}$ (n = 5 for Saline and n = 5 for *H1N1*). All bars in the graphs represent the mean ± s.e.m. Statistical comparisons were performed using one-way ANOVA with Tukey's multiple comparisons test (**c**). Source data are provided as a Source Data file.

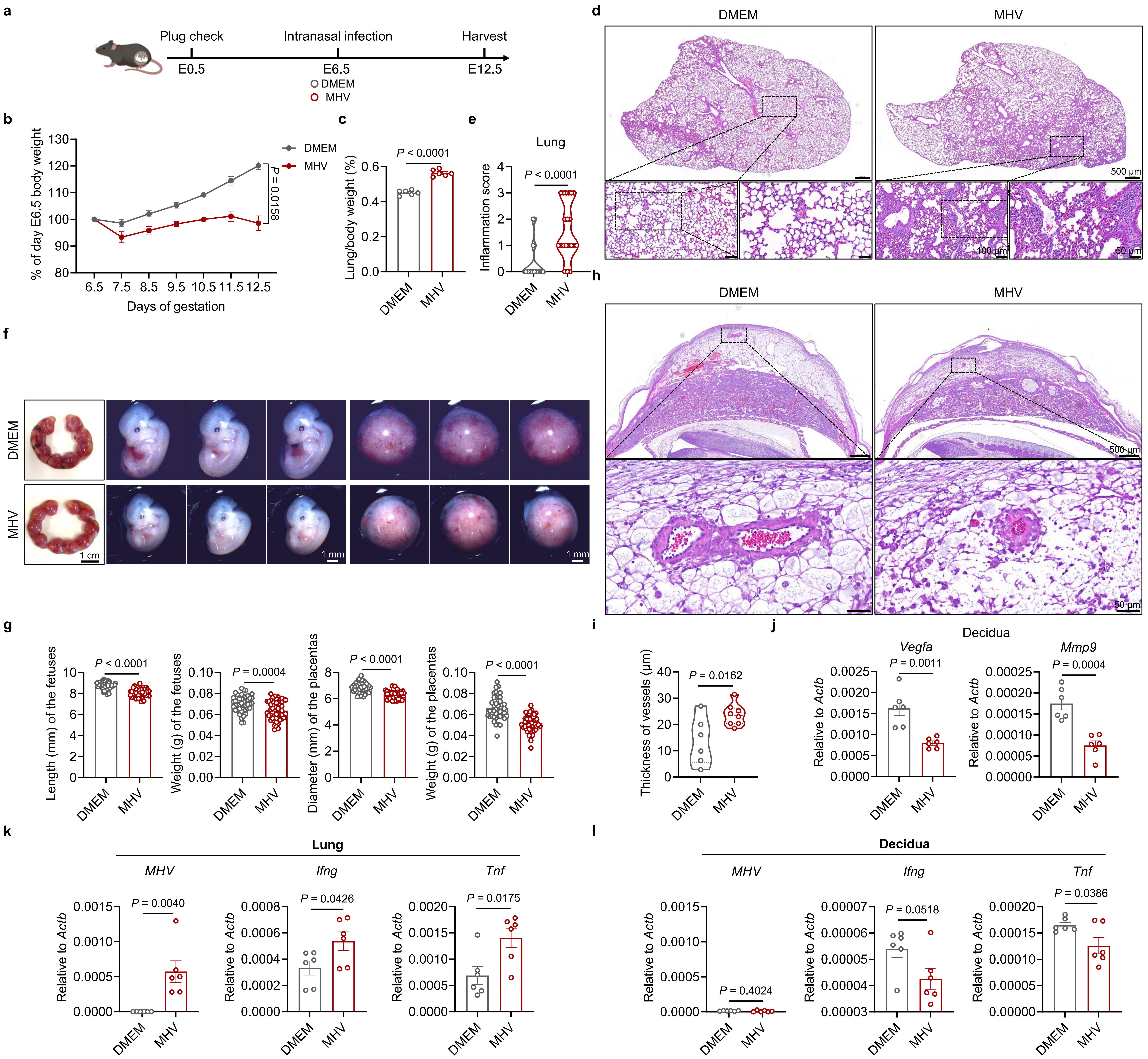

Supplementary Fig. 9 | Intranasal MHV inoculation during early pregnancy causes lung inflammation and intrauterine growth restriction, related to Fig. 7. a Schematic diagram illustrating the timeline of MHV infection in pregnant mice (Created in BioRender. Ding, X. (2026) https://BioRender.com/lfokc6w). **b** Changes in body weight of pregnant mice infected with either DMEM (n = 6) or MHV (n = 6). **c-l** Maternal lungs and deciduas were collected at embryonic day 12.5 (E12.5) for analysis. **c** Statistical analysis of the ratio of lung tissue mass to mouse body weight in pregnant mice infected with either DMEM (n = 6) or MHV (n = 6). **d, e** Representative images of H&E staining of lung sections infected with either DMEM or MHV (**d**) and the inflammation score for lung sections (**e**). scale bar, 50 μm. **f, g** Representative images of the uterus, fetuses, and placentas (**f**) and statistical analysis of the length and weight of fetuses and the diameter and weight of placentas (**g**) infected with either DMEM (n = 44 for length of fetuses, n = 44 for weight of fetuses, n = 43 for diameter of placentas, and n = 43 for weight of placentas) or MHV (n = 49 for length of fetuses, n = 49 for weight of fetuses, n = 49 for diameter of placentas, and n = 50 for weight of placentas). scale bar, 1 cm. **h, i** Representative images of H&E staining of vessels in decidua sections (**h**) and statistical analysis of vessel thickness (**i**) infected with either DMEM (n = 6) or MHV (n = 8), and. scale bar, 50 μm. **j** qPCR analysis of *Vegfa* and *Mmp9* expression in deciduas infected with either DMEM (n = 6) or MHV (n = 6). **k, l** qPCR analysis of *MHV*, *Ifng*, and *Tnf* expression in lungs (**k**) and deciduas (**l**) infected with either DMEM (n = 6) or MHV (n = 6). Results are representative of two or three independent experiments. All bars in the graphs represent the mean ± s.e.m. Statistical comparisons were performed using a two-tailed unpaired Student's t-test (**b**, **c**, **e**, **g**, **i**, **j**, **k** and **l**). Source data are provided as a Source Data file.

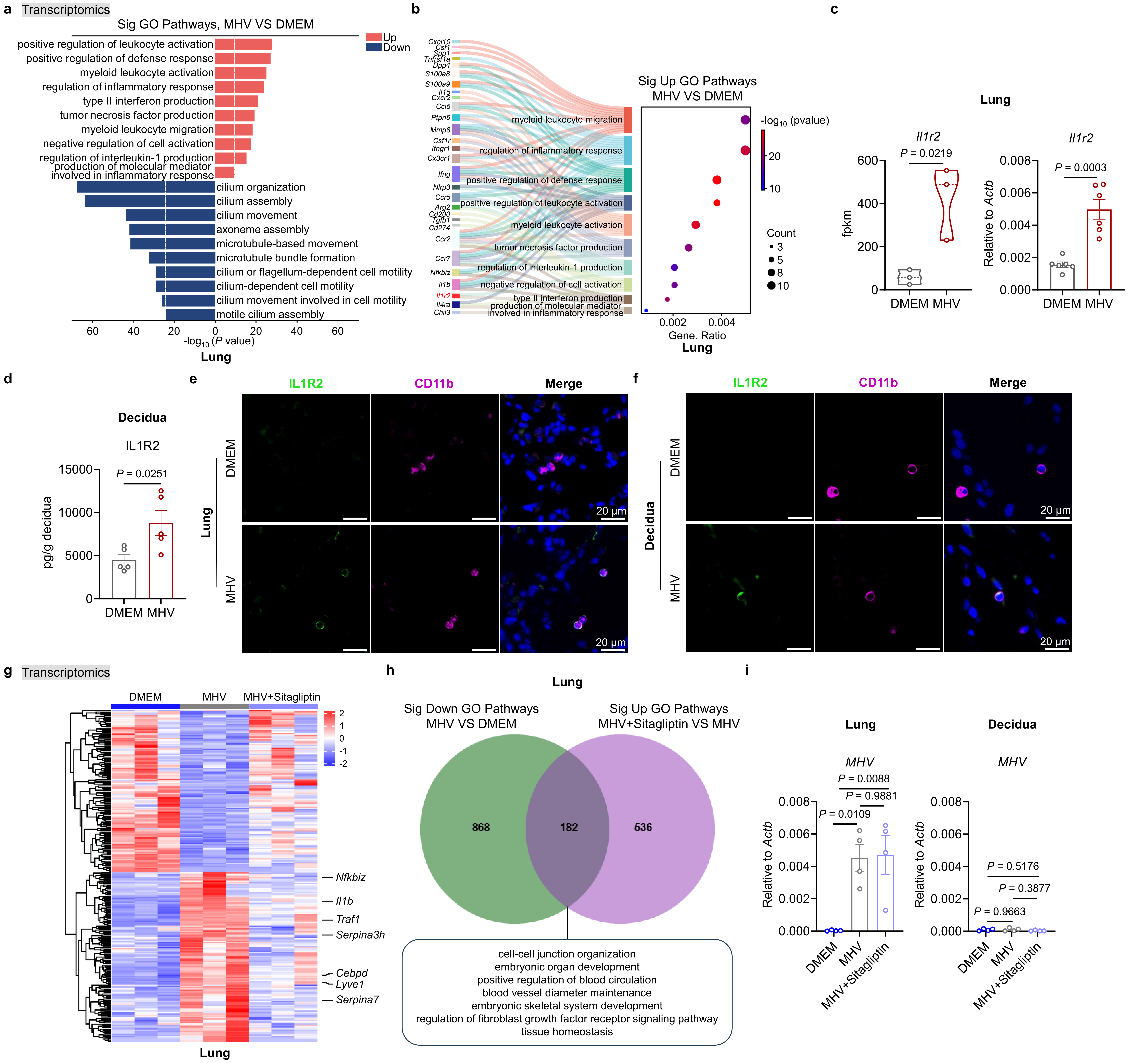

Supplementary Fig. 10 | Intranasal MHV inoculation results in the accumulation of IL1R2 and inhibition of DPP4 alleviates lung inflammation, related to Fig. 7. a-i Maternal lungs and deciduas were collected at embryonic day 12.5 (E12.5) for analysis. **a** Gene Ontology (GO) analysis of differentially expressed genes in lungs infected with either DMEM (n = 3) or MHV (n = 3). **b** Sankey dot plot analysis for pathway enrichment of differentially expressed genes in lungs infected with either DMEM (n = 3) or MHV (n = 3). **c** Fpkm values and qPCR of *Il1r2* expression in lungs infected with either DMEM (n = 6) or MHV (n = 6). **d** ELISA analysis of IL1R2 in deciduas infected with either DMEM (n = 5) or MHV (n = 5). **e, f** Representative images of immunofluorescence staining of CD11b and IL1R2 in lung sections (**e**) and decidua sections (**f**) infected with either DMEM or MHV. scale bar, 20 μm. **g** Gene expression heatmap in lungs infected with either DMEM (n = 3) or MHV alone (n = 3), or treated with sitagliptin (n = 3). **h** Venn diagram showing the shared GO pathways downregulated in MHV infected lungs and upregulated after treatment with sitagliptin. **i** qPCR analysis of *MHV* in lungs and deciduas infected with either DMEM (n = 4) or MHV alone (n = 4), or treated with sitagliptin (n = 4). Results are representative of two or three independent experiments. All bars in the graphs represent the mean ± s.e.m. Statistical comparisons were performed using a two-tailed unpaired Student's t-test (**c** and **d**) and one-way ANOVA with Tukey's multiple comparisons test (**i**). Source data are provided as a Source Data file.

Supplementary Table 1. PCR primers used for genotyping of *Il1r2*$^{-/-}$ mice, related to the methods

Name	Forward primers (5 -3)	Reverse primers (5 -3)
PCR1	TTATCTTCTAGCGACTGGAGTCAG	AGTCCTTACAGATAAGAGCCAGATG
PCR2	TTATCTTCTAGCGACTGGAGTCAG	TCTAGTCTTCCAAGACACCTGC

Supplementary Table 2. Primers used for real-time quantitative PCR, related to the methods

Genes	Forward primers (5 -3)	Reverse primers (5 -3)
Mouse *Actb*	TGACGTTGACATCCGTAAAGACC	CTCAGGAGGAGCAATGATCTTGA
Mouse *Il6*	GATGCTACCAAACTGGATATAATC	GGTCCTTAGCCACTCCTTCTGTG
Mouse *Tnf*	CAGGCGGTGCCTATGTCTC	CGATCACCCCGAAGTTCAGTAG
Mouse *Il1b*	AATGCCACCTTTTGACAGTGA	GATGTGCTGCTGCGAGATTT
H1N1	GGACTGCAGCGTAGACGCTT	CATCCTGTTGTATATGAGGCCCAT
Mouse *Ifna*	TACTCAGCAGACCTTGAACCT	CAGTCTTGGCAGCAAGTTGAC
Mouse *Ifnb*	TGGGTGGAATGAGACTATTGTTG	CTCCCACGTCAATCTTTCCTC
Mouse *Usp18*	TCTGTTCACCATCTGGACGC	CAAGGCATCCTCCAGGGTTT
Mouse *Vegfa*	ACGAAAGCGCAAGAAATCCC	GCAACGCGAGTCTGTGTTTT
Mouse *Mmp9*	CTGGACAGCCAGACACTAAAG	CTCGCGGCAAGTCTTCAGAG
Mouse *Il1r2*	CCCCTGGAGACAATACCAGC	TTAGCCAACCACCACACAATG
Mouse *Il1rap*	AGGAATTTGTGCTGCTGACG	CTTCCACGGTATTTCCCCCA
Mouse *Il1rn*	GGGGACCCTACAGTCACCTAAT	GCATCTTGCAGGGTCTTTTCC
MHV	TATAAGAGTGATTGGCGTCC	GAGTAATGGGGAACCACACT
Mouse *Ifng*	ATGAACGCTACACACTGCATC	CCATCCTTTTGCCAGTTCCTC

Supplementary Table 3. Antibodies used for flow cytometry analysis, related to the methods

Antibodies	Source	Identifier	Clone	Dilution
PE anti-mouse CD121b Antibody	BD	554450; PRID: AB_395399	4E2	1:100
FITC anti-mouse Ly-6C Antibody	BD	553104; PRID: AB_394628	AL-21	1:100
BV510 anti-mouse Ly-6G Antibody	Biolegend	127633; PRID: AB_2562937	1A8	1:100
PerCP-Cy5.5 anti-mouse Ly-6G/Ly-6C (Gr-1) Antibody	Biolegend	108428; PRID: AB_893558	RB6-8C5	1:100
BV605 anti-mouse F4/80 Antibody	BD	743281; PRID: AB_2741399	T45-2342	1:100
APC Mouse anti-Mouse CD45.2	BD	558702; PRID: AB_1645215	104	1:100
PerCP/Cyanine5.5 anti-mouse/human CD11b Antibody	Biolegend	101228; PRID: AB_893232	M1/70	1:100
PE-Cy7 anti-mouse CD45 Antibody	Biolegend	103114; PRID: AB_312979	30-F11	1:100
BV421 anti-mouse CD11b Antibody	Biolegend	101236; PRID: AB_11203704	M1/70	1:100

Supplementary Table 4. Antibodies used for immunohistochemistry and immunofluorescence, related to the methods

Antibodies	Source	Identifier	Clone	Dilution
F4/80 (D2S9R) XP® Rabbit mAb	CST	70076; PRID: AB_2799771	D2S9R	1:500
Ly-6G (E6Z1T) Rabbit mAb	CST	87048; PRID: AB_2909808	E6Z1T	1: 300
Anti-Cytokeratin 18 antibody	Abcam	ab181597; PRID: AB_2922417	EPR17347	1:800
Anti-IL-1R-2 antibody	Abcam	ab212208; PRID: AB_3675298		1:200
Anti-CD11b antibody	Abcam	ab133357; PRID: AB_2650514	EPR1344	1:4000
Anti-Influenza A Virus Nucleoprotein antibody	Abcam	ab20343; RRID: AB_445525	AA5H	1:1000
