# Supplementary material for: Dipeptidylpeptidase 4 inhibition attenuates gestational pathologies via immune homeostasis restoration in the pulmonary-uterine axis
Source: Nat Commun. 2026 Feb 17;17:2851. doi: 10.1038/s41467-026-69620-9 (PMC13021972; doi:10.1038/s41467-026-69620-9)
Supplement: Supplementary file 2 — Reporting Summary [file 41467_2026_69620_MOESM2_ESM.pdf]

nature portfolio

Corresponding author(s): Binqing Fu, Haiming Wei

Last updated by author(s): Nov 26, 2025

Reporting Summary

Nature Portfolio wishes to improve the reproducibility of the work that we publish. This form provides structure for consistency and transparency in reporting. For further information on Nature Portfolio policies, see our Editorial Policies and the Editorial Policy Checklist.

Statistics

For all statistical analyses, confirm that the following items are present in the figure legend, table legend, main text, or Methods section.

n/a	Confirmed	
☐	☒	The exact sample size (*n*) for each experimental group/condition, given as a discrete number and unit of measurement
☐	☒	A statement on whether measurements were taken from distinct samples or whether the same sample was measured repeatedly
☐	☒	The statistical test(s) used AND whether they are one- or two-sided<br>*Only common tests should be described solely by name; describe more complex techniques in the Methods section.*
☒	☐	A description of all covariates tested
☒	☐	A description of any assumptions or corrections, such as tests of normality and adjustment for multiple comparisons
☐	☒	A full description of the statistical parameters including central tendency (e.g. means) or other basic estimates (e.g. regression coefficient) AND variation (e.g. standard deviation) or associated estimates of uncertainty (e.g. confidence intervals)
☐	☒	For null hypothesis testing, the test statistic (e.g. *F*, *t*, *r*) with confidence intervals, effect sizes, degrees of freedom and *P* value noted<br>*Give P values as exact values whenever suitable.*
☒	☐	For Bayesian analysis, information on the choice of priors and Markov chain Monte Carlo settings
☒	☐	For hierarchical and complex designs, identification of the appropriate level for tests and full reporting of outcomes
☒	☐	Estimates of effect sizes (e.g. Cohen's *d*, Pearson's *r*), indicating how they were calculated

Our web collection on statistics for biologists contains articles on many of the points above.

Software and code

Policy information about availability of computer code

Data collection	The Flow cytometric assays: Celesta flow cytometer (BD Biosciences, San Jose, CA, USA)<br>The hematoxylin and eosin, immunohistochemistry and immunofluorescence assays: MIDI digital slide scanner (3D HISTECH, Budapest, Hungary)<br>Quantitative analysis of immunohistochemistry images: ImageJ (Bethesda, MD, USA, version 1.54d,).<br>The qPCR assays: LightCycler 96 Real-Time PCR System (Roche, Mannheim, Germany)<br>The RNA-seq assays: Novaseq 6000 Platform ((Illumina, San Diego, CA, USA)<br>The Flow sorting assays: Thermo Fisher Scientific Bigfoot Full-Spectrum Flow Cytometer (Waltham, MA, USA)<br>The Single-cell RNA sequencing assays: Singleron Matrix NEO™ Single Cell Processing System and GEXSCOPE® Single Cell RNA Library Kits (Singleron, Nanjing, China); Illumina NovaSeq 6000 system (San Diego, CA, USA); CeleScope pipeline (v1.9.0)<br>The Cytokine antibody array assays: Quantibody® QAM-TH17-1 Array (RayBiotech, Norcross, GA, USA)<br>The Proteome analysis assays: Q Exactive HF-X Hybrid Quadrupole-Orbitrap mass spectrometer (Thermo Fisher Scientific, Waltham, MA, USA)
Data analysis	Statistical analysis: Prism 8.0 (GraphPad, San Diego, CA, USA)<br>Flow cytometric analysis: FlowJo (Version10.1 for Windows)<br>Hematoxylin and eosin, immunohistochemistry and immunofluorescence analysis: CaseViewer (3DHISTECH, Budapest, Hungary)<br>qPCR analysis: LightCycler 96 SW 1.1 software (Roche, Basel, Switzerland)<br>RNA-seq analysis: DESeq2 package (v1.42.0) and clusterProfiler package (v4.8.1) in R (v4.3.1, R Core Team, 2023)<br>Single-cell RNA sequencing analysis: Scanpy package (v1.9.0) in Python (v3.9)<br>Cytokine antibody array analysis: GenePix Pro (Molecular Devices, Sunnyvale, CA, USA)

Proteome analysis analysis: DIA-NN (version 1.7.0)

For manuscripts utilizing custom algorithms or software that are central to the research but not yet described in published literature, software must be made available to editors and reviewers. We strongly encourage code deposition in a community repository (e.g. GitHub). See the Nature Portfolio guidelines for submitting code & software for further information.

Data

Policy information about availability of data

All manuscripts must include a data availability statement. This statement should provide the following information, where applicable:

- Accession codes, unique identifiers, or web links for publicly available datasets
- A description of any restrictions on data availability
- For clinical datasets or third party data, please ensure that the statement adheres to our policy

The raw sequencing data generated in this study have been deposited in the Genome Sequence Archive (GSA) at the National Genomics Data Center under accession number CRA023974 and are publicly accessible at https://ngdc.cncb.ac.cn/gsa/browse/CRA023974. The proteomics data generated in this study have been deposited in the OMIX repository at the National Genomics Data Center under accession number OMIX013325. All data are included in the Supplementary Information or available from the authors, as are unique reagents used in this Article. The raw numbers for charts and graphs are available in the Source Data file whenever possible. Source data are provided with this paper.

Research involving human participants, their data, or biological material

Policy information about studies with human participants or human data. See also policy information about sex, gender (identity/presentation), and sexual orientation and race, ethnicity and racism.

Reporting on sex and gender	Not applicable (no human participants, data, or biological materials involved)
Reporting on race, ethnicity, or other socially relevant groupings	Not applicable (no human participants, data, or biological materials involved)
Population characteristics	Not applicable (no human participants, data, or biological materials involved)
Recruitment	Not applicable (no human participants, data, or biological materials involved)
Ethics oversight	Not applicable (no human participants, data, or biological materials involved)

Note that full information on the approval of the study protocol must also be provided in the manuscript.

Field-specific reporting

Please select the one below that is the best fit for your research. If you are not sure, read the appropriate sections before making your selection.

[x] Life sciences [ ] Behavioural & social sciences [ ] Ecological, evolutionary & environmental sciences

For a reference copy of the document with all sections, see nature.com/documents/nr-reporting-summary-flat.pdf

Life sciences study design

All studies must disclose on these points even when the disclosure is negative.

Sample size	No statistical methods were used to predetermine sample size. Sample sizes were indicated in the figure legends. Sample sizes were chosen based on our preliminary experiments or previous publications.
Data exclusions	No data were excluded from the analyses.
Replication	The number of biological replicates and independent experiments were included in Figure legends.
Randomization	Samples were randomly distributed into groups.
Blinding	Virus infections, in vivo drug treatment and measurement of body weight were performed in a blinded manner. Other experiments were not performed in a blinded manner, as the investigators were aware of the group allocation due to the need for clarity regarding treatment. groups during the experimental procedures.

Reporting for specific materials, systems and methods

We require information from authors about some types of materials, experimental systems and methods used in many studies. Here, indicate whether each material, system or method listed is relevant to your study. If you are not sure if a list item applies to your research, read the appropriate section before selecting a response.

Materials & experimental systems

n/a	Involved in the study
	☒ Antibodies
	☒ Eukaryotic cell lines
☒	Palaeontology and archaeology
	☒ Animals and other organisms
☒	Clinical data
☒	Dual use research of concern
☒	Plants

Methods

n/a	Involved in the study
☒	ChIP-seq
	☒ Flow cytometry
☒	MRI-based neuroimaging

Antibodies

Antibodies used	Antibodies SOURCE Catalog #<br>PE anti-mouse CD121b Antibody, BD, 554450;<br>FITC anti-mouse Ly-6C Antibody, BD, 553104;<br>BV510 anti-mouse Ly-6G Antibody, Biolegend, 127633;<br>PerCP-Cy5.5 anti-mouse Ly-6G/Ly-6C (Gr-1) Antibody, Biolegend, 108428;<br>BV605 anti-mouse F4/80 Antibody, BD, 743281;<br>APC anti-Mouse CD45.2 Antibody, BD, 558702;<br>PerCP/Cyanine5.5 anti-mouse/human CD11b Antibody, Biolegend, 101228;<br>PE-Cy7 anti-mouse CD45 Antibody, Biolegend, 103114;<br>BV421 anti-mouse CD11b Antibody, Biolegend, 101236;<br>F4/80 (D2S9R) XP® Rabbit mAb, CST, 70076;<br>Ly-6G (E6Z1T) Rabbit mAb, CST, 87048;<br>Anti-Cytokeratin 18 antibody, Abcam, ab181597;<br>Anti-IL-1R-2 antibody, Abcam, ab212208;<br>Anti-CD11b antibody, Abcam, ab133357;<br>Anti-Influenza A Virus Nucleoprotein antibody, Abcam, ab20343.
Validation	The antibodies used in the study were validated by the corresponding manufactures. Commercial antibodies were used in accordance with the manufactures' recommendation as provided on their official websites. The validation of the species and application of all the primary antibodies by the manufactures are provided as follows.<br>PE anti-mouse CD121b Antibody, species: Mouse, applications: FC. https://www.bdbiosciences.com/zh-cn/products/reagents/flowcytometry-reagents/research-reagents/single-color-antibodies-ruo/pe-rat-anti-mouse-cd121b.554450?tab=product_details.<br>FITC anti-mouse Ly-6C Antibody, species: Mouse, applications: FC. https://www.bdbiosciences.com/zh-cn/products/reagents/flowcytometry-reagents/research-reagents/single-color-antibodies-ruo/fitc-rat-anti-mouse-ly-6c.553104?tab=product_details<br>BV510 anti-mouse Ly-6G Antibody, species: Mouse, applications: FC. https://www.biolegend.com/en-gb/products/brilliantviolet-510-anti-mouse-ly-6g-antibody-9121.<br>PerCP-Cy5.5 anti-mouse Ly-6G/Ly-6C (Gr-1) Antibody, species: Mouse, applications: FC. https://www.biolegend.com/en-gb/products/percp-cyanine5-5-anti-mouse-ly-6g-ly-6c-gr-1-antibody-4286.<br>BV605 anti-mouse F4/80 Antibody, species: Mouse, applications: FC. https://www.bdbiosciences.com/zh-cn/products/reagents/flow-cytometry-reagents/research-reagents/single-color-antibodies-ruo/bv605-rat-anti-mouse-f4-80.743281?tab=product_details.<br>APC anti-Mouse CD45.2 Antibody, species: Mouse, applications: FC. https://www.bdbiosciences.com/zh-cn/products/reagents/flow-cytometry-reagents/research-reagents/single-color-antibodies-ruo/apc-mouse-anti-mouse-cd45-2.558702?tab=product_details.<br>PerCP/Cyanine5.5 anti-mouse/human CD11b Antibody, species: Mouse, Human, Cynomolgus, Rhesus, applications: FC. https://www.biolegend.com/en-gb/products/percp-cyanine5-5-anti-mouse-human-cd11b-antibody-4257.<br>PE-Cy7 anti-mouse CD45 Antibody, species: Mouse, applications: FC. https://www.biolegend.com/en-gb/products/pe-cyanine7-antimouse-cd45-antibody-1903.<br>BV421 anti-mouse CD11b Antibody, species: Mouse, Human, Cynomolgus, Rhesus, applications: FC. https://www.biolegend.com/en-gb/products/brilliant-violet-421-anti-mouse-human-cd11b-antibody-7163.<br>F4/80 (D2S9R) XP® Rabbit mAb, species: Mouse, applications: WB, IP, IHC. https://www.cellsignal.cn/products/primary-antibodies/f4-80-d2s9r-xp-rabbit-mab/70076.<br>Ly-6G (E6Z1T) Rabbit mAb, species: Mouse, applications: WB, IHC. https://www.cellsignal.cn/products/primary-antibodies/ly-6g-e6z1t-rabbit-monoclonal-antibody/87048.<br>Anti-Cytokeratin 18 antibody, species: Human, mouse, Rat, applications: WB, IHC-P. https://www.abcam.cn/products/primaryantibodies/cytokeratin-18-antibody-epr17347-ab181597.html.<br>Anti-IL-1R-2 antibody, species: Human, mouse, Rat, applications: WB, IHC-P. https://www.abcam.cn/products/primary-antibodies/il-1r-2-antibody-ab212208.html.<br>Anti-CD11b antibody, species: Human, mouse, Rat, applications: WB, IHC-P. mIHC.https://www.abcam.cn/products/primaryantibodies/cd11b-antibody-epr1344-ab133357.html.<br>Anti-Influenza A Virus Nucleoprotein antibody, species: Influenza A, applications: ICC/IF, IHC-P. https://www.abcam.cn/products/primary-antibodies/influenza-a-virus-nucleoprotein-antibody-aa5h-ab20343.

Eukaryotic cell lines

Policy information about cell lines and Sex and Gender in Research

Cell line source(s)	MDCK was provided by cooperator.
Authentication	We used morphology analysis with microscopy to authenticate cell line.
Mycoplasma contamination	We had not tested mycoplasma contamination.
Commonly misidentified lines (See ICLAC register)	We had not used any commonly misidentified lines.

Animals and other research organisms

Policy information about studies involving animals; ARRIVE guidelines recommended for reporting animal research, and Sex and Gender in Research

Laboratory animals	Seven-week-old female and eight-week-old male C57BL/6 mice were purchased from Shanghai SLAC Laboratory Animal Company (stock number: scb0105). Il1r2+/- mice were obtained from GemPharmatech and bred to generate Il1r2+/+ and Il1r2-/- genotypes (stock number: T005564). Genotyping was performed via standard PCR using primers listed in Supplementary Table 1. All mice were maintained on a C57BL/6 background. Littermate and cage-mate controls were used throughout the study. Mice were housed under specific pathogen-free (SPF) conditions, with a 12-hour light/dark cycle, ambient temperature of 20–26°C, and relative humidity of 50–70%. All animals were fed an irradiation-sterilized mouse diet (WQ JXBIO-TECHNOLOGY).
Wild animals	No wild animals were used.
Reporting on sex	All experiments only used pregnant female mice after mating.
Field-collected samples	No field-collected samples were used.
Ethics oversight	Experimental protocols were approved by the Ethics Committee of the University of Science and Technology of China (USTCACUC27120122083) and adhered to the National Animal Research Guidelines (China).

Note that full information on the approval of the study protocol must also be provided in the manuscript.

Plants

Seed stocks	*Report on the source of all seed stocks or other plant material used. If applicable, state the seed stock centre and catalogue number. If plant specimens were collected from the field, describe the collection location, date and sampling procedures.*
Novel plant genotypes	*Describe the methods by which all novel plant genotypes were produced. This includes those generated by transgenic approaches, gene editing, chemical/radiation-based mutagenesis and hybridization. For transgenic lines, describe the transformation method, the number of independent lines analyzed and the generation upon which experiments were performed. For gene-edited lines, describe the editor used, the endogenous sequence targeted for editing, the targeting guide RNA sequence (if applicable) and how the editor was applied.*
Authentication	*Describe any authentication procedures for each seed stock used or novel genotype generated. Describe any experiments used to assess the effect of a mutation and, where applicable, how potential secondary effects (e.g. second site T-DNA insertions, mosiacism, off-target gene editing) were examined.*

Flow Cytometry

Plots

Confirm that:

- [x] The axis labels state the marker and fluorochrome used (e.g. CD4-FITC).
- [x] The axis scales are clearly visible. Include numbers along axes only for bottom left plot of group (a 'group' is an analysis of identical markers).
- [x] All plots are contour plots with outliers or pseudocolor plots.
- [x] A numerical value for number of cells or percentage (with statistics) is provided.

Methodology

Sample preparation	For lung leukocytes, lung tissue was minced and digested with 1 mg/mL collagenase type I (Sigma-Aldrich) in RPMI 1640 medium (VivaCell, Shanghai) at 37 °C for 1 hour. Digests were filtered through nylon mesh, followed by density gradient centrifugation in 40% Percoll at 750 × g for 20 minutes. Red blood cells were lysed, and leukocytes were collected. Peripheral blood mononuclear cells (PBMCs) were isolated after red blood cell lysis. For decidual leukocytes, tissues were harvested at E12.5 after embryos were carefully removed. The decidua was minced and digested in 2 mg/mL collagenase type IV (Sigma-Aldrich) in RPMI 1640 at 37 °C for 45 minutes. The resulting suspensions were filtered, and leukocytes were isolated using the

	same Percoll-based protocol. Isolated cells were suspended in phosphate-buffered saline (PBS) containing 1% fetal bovine serum, blocked with Fc receptor antibodies, and stained with fluorophore-conjugated antibodies for 30 minutes at 4 °C in the dark.
Instrument	Celesta flow cytometer (BD Biosciences)
Software	FlowJo (version 10.1) was used for flow cytometry data analysis.
Cell population abundance	There was no sorting performed.
Gating strategy	FSC-A/FSC-H plots were used to determine singlet gates. Additional gating was performed as described in the Supplementary materials (Supplementary Fig. 6a).

☒ Tick this box to confirm that a figure exemplifying the gating strategy is provided in the Supplementary Information.
